# Nanocarrier-Based Drug Delivery for Melanoma Therapeutics

**DOI:** 10.3390/ijms22041873

**Published:** 2021-02-13

**Authors:** Mingming Song, Chang Liu, Siyu Chen, Wenxiang Zhang

**Affiliations:** State Key Laboratory of Natural Medicines and School of Life Science and Technology, China Pharmaceutical University, Nanjing 211198, China; 1831030122@stu.cpu.edu.cn (M.S.); changliu@cpu.edu.cn (C.L.)

**Keywords:** melanoma, drug delivery systems, immunotherapy, gene therapy, photodynamic therapy, combination therapy

## Abstract

Melanoma, as a tumor cell derived from melanocyte transformation, has the characteristics of malignant proliferation, high metastasis, rapid recurrence, and a low survival rate. Traditional therapy has many shortcomings, including drug side effects and poor patient compliance, and so on. Therefore, the development of an effective treatment is necessary. Currently, nanotechnologies are a promising oncology treatment strategy because of their ability to effectively deliver drugs and other bioactive molecules to targeted tissues with low toxicity, thereby improving the clinical efficacy of cancer therapy. In this review, the application of nanotechnology in the treatment of melanoma is reviewed and discussed. First, the pathogenesis and molecular targets of melanoma are elucidated, and the current clinical treatment strategies and deficiencies of melanoma are then introduced. Following this, we discuss the main features of developing efficient nanosystems and introduce the latest reports in the literature on nanoparticles for the treatment of melanoma. Subsequently, we review and discuss the application of nanoparticles in chemotherapeutic agents, immunotherapy, mRNA vaccines, and photothermal therapy, as well as the potential of nanotechnology in the early diagnosis of melanoma.

## 1. Introduction

Cutaneous melanoma is a type of skin cancer whose incidence is increasing significantly worldwide [1,2,3]. Although melanoma occurs infrequently, accounting for less than 5% of skin cancer, it is highly aggressive and accounts for more than 75% of all skin cancer deaths [4,5,6,7,8]. Furthermore, melanoma is the third most common source of brain metastases after lung and breast cancer, with more than 60% of patients with metastatic melanoma having or developing brain metastases during their onset [9]. The early detection of melanoma is a key factor for melanoma therapy [10,11]. Although an earlier diagnosis has been documented with better outcomes, one-fifth of deaths counterintuitively occur in patients who are initially presenting with early disease [12]. Melanoma is a complex disease with a poor prognosis. In clinical cases, melanoma is diagnosed in the last stages and metastatic forms, and it is known that individual cells can switch from a proliferative state to an invasive state [13,14,15]. These can lead to patients with melanoma exhibiting a hard response to the current therapeutic approaches.

The continuous progress in science technology and understanding of cell and tumor biology has improved cancer treatment. However, the treatment results for melanoma are still disappointing because, in most cases, the treatment is ineffective [16]. Drug resistance is an important characteristic of melanoma, resulting in the lack of effectiveness of current drug treatments [17,18,19,20,21]. Surgery [22,23], chemotherapy [24,25], and immunotherapy [26,27] are the most commonly approaches. However, these treatments are largely limited by an advanced cancer diagnosis, off-target drug delivery and concentration, systemic toxicity, and drug-induced undesirable side effects. When melanoma is detected at an early stage (stage I and II), it can be cured by surgical removal of the tumor [28]. This treatment method can effectively prevent the early metastasis of melanoma [29]. However, in most advanced melanomas, surgical treatment still fails to achieve the expected value. Chemotherapy is currently the most commonly used cancer treatment as a single or drug combination therapy, increasing the survival time for cancer patients [30,31,32]. However, the application of anticancer drugs still have serious limitations that often compromise the effectiveness and continuity of treatment. Common chemotherapeutic agents trigger the cell breakdown by inducing DNA damage and strand breaks, interfering with DNA repair and microtubule function (especially taxanes) [33,34]. These chemotherapy drugs can not only kill tumor cells but also damage normal tissue cells. At the same time, another worrying obstacle related to cancer treatment is tumor cell resistance. Such resistances may due to internal factors, including mutations [35,36], gene amplification [37,38], deletions [39,40], and chromosomal rearrangements [41,42], or external factors, such as pH [43], hypoxia [44], and paracrine signaling interactions with stromal cells [45,46]. Currently, the main clinical drugs employed for melanoma treatment include doxorubicin (DOX) [47], vemurafenib [48], and paclitaxel (PTX) [49], but these drugs lack specificity for tumor sites, and melanoma cells often develop drug resistance to these drugs. Furthermore, these drugs seem to have no obvious therapeutic effect and even have serious side effects. Moreover, melanoma is one of the most sensitive malignant tumors to immune regulation [48]. Despite decades of trials of vaccines, cytokines, and cell therapies, it has been shown to be meaningful in a small proportion of patients with metastatic disease. To date, the metastasis probability of melanoma is still high, and this effect is partly due to tumor-driven immunosuppression. At present, most patients encounter multiple challenges in treatment, including severe side effects and drug resistance. To overcome these challenges, there is an urgent need to develop new treatment methods that can be combined with current therapies to help improve clinical treatment. Nanomedicine is a promising strategy that can improve the efficacy of drugs by increasing the concentration of drugs at tumor sites, thereby improving the clinical effects of cancer treatment [50,51,52].

**Figure 1:**
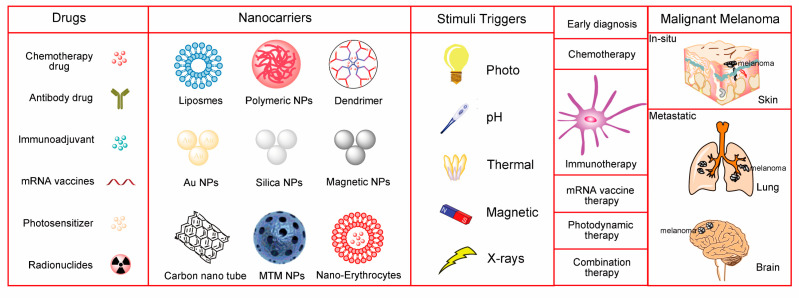
Nanoparticle-based drug delivery for melanoma therapeutics. Factors that are considered in a sensible design strategy should include the composition of the nanocarrier core, targeting ligands, stimulation–responsive triggers for specific site release of cargo, and expected therapeutic outcomes. Latest therapies for primary melanoma and metastases include the nanocarrier-based target delivery of chemotherapeutic drugs, antibodies, and mRNA; nanocarrier-induced immune regulation to activate anticancer immune responses; and nanocarrier-activated photothermal and radiotherapy for in-situ/metastatic melanoma, NPs: nanoparticles; MTM: core-shell MnO_2_@TiO_2_.

In this review, we discuss the challenges of treating metastatic melanoma and the latest advances in nanoparticles in overcoming these challenges. A special focus is placed on the latest treatments for primary melanoma and metastases, including the nanocarrier-based target delivery of chemotherapeutic drugs, antibodies, and mRNA; nanocarrier-induced immune regulation to activate anticancer immune responses; and nanocarrier-activated photothermal and radiotherapy for in-situ/metastatic melanoma (Figure 1). Finally, we provide insights on the design and use of nanoparticles to further promote the clinical application of melanoma imaging and treatment.

## 2. Medical Nanomaterials in Tumor Therapy

The application of nanotechnology has greatly improved clinical practice in the diagnosis, treatment, and management of cancer. Nanotechnology also provides strategies for targeted delivery of drugs, genes, and proteins to tumors, thereby reducing their nonspecific accumulation in peripheral tissues [53,54,55]. Currently, the major medical nanomaterials include organic (e.g., liposome, polymeric nanoparticles, and dendrimer) and inorganic (e.g., magnetic nanoparticles, carbon nanoparticles, gold nanoparticles, and silica nanoparticles) nanomaterials [56,57,58], which are used in the diagnosis and treatment of various cancers. Liposomes are amphoteric lipid bilayers with a hydrophilic core and a hydrophobic outer shell [59]. Because of such a unique structure, liposomes can encapsulate not only hydrophilic drugs but also encapsulate hydrophobic drugs [25,60,61]. Liposomes are more biocompatible than other synthetic materials due to their similar composition to cell membranes [62]. Liposomes protect drugs from degradation and prevent premature exposure to the environment, thereby preventing drug enrichment in non-target organs [60]. However, the challenge for the development of liposomes as drug carriers is how to control the specific distribution and clearance of liposomes in vivo. Polymer nanoparticles are particles with a diameter of less than 1 μm prepared from natural (proteins, chitosan, cyclodextrin, and starch) [61,63,64,65] and synthetic (polyethylene glycol, PEG and Poly (d, l-lactic-coglycolic acid), PLGA) [66] polymers. Polymer nanoparticles can be obtained with different properties and different release characteristics by forming matrix-type or reservoir-type structures according to different preparation methods. Polymer nanoparticles can also improve the specificity of drug action by altering the tissue distribution and pharmacokinetics of drugs in the patient and are, therefore, considered as promising drug carriers. At present, the research of polymer nanocarriers mainly focuses on the elucidation of the mechanism of action, environmental reaction, activity localization, and composite materials. Importantly, the key feature of polymer nanoparticles as drug carriers is that they can be surface functionalized to target tumor tissues or cells actively and stimulate responsiveness and control the release of drugs [67]. Actively targeting nanoparticles to the site of action is based on tumors. Li et al. successfully synthesized chitosan-based polymer nanoparticles by ion gel method, which has a high capacity to loaded DOX and human thrombin [68]. At the same time, the tumor homing pentapeptide with sequence CREKA was grafted onto the surface of nanoparticles to produce nanoparticles with an active tumor tissue targeting ability [69].

In addition, due to the specific physical and chemical properties of inorganic nanoparticles, which include non-metal and metal nanoparticles, such as carbon nanotubes [70], gold nanoparticles [71], magnetic nanoparticles [72], and silicon nanoparticles [73], can improve the delivery efficiency of drugs and the early diagnosis of tumors. In the drug delivery field, carbon nanotubes are cylinders composed of several coaxial graphite layers with a diameter of nanometers [74]. Due to their thermal conductivity and optical properties, they have become a popular candidate material for killing cancer cells through local hyperthermia [75]. Furthermore, carbon nanotubes can be specifically taken up by tumor cells through the functional modification of tumor-specific ligands (folate, FA) [76] or antibodies (monoclonal antibodies) [77]. In the bioimaging field, due to the high electron density of metal nanoparticles, these particles are now used to evaluate the interaction of different bio-specific molecules and detect their specific membrane antigens for the early diagnosis of tumors [78]. In addition, in this review, we also summarized the relevant research on medical nanomaterials with different properties used in the treatment of melanoma in the last three years (Table 1). However, early experimental results show that inorganic nanomaterials, including gold nanoparticles and silicon nanoparticles, have toxic side effects [79,80,81]. In summary, different nanomaterials play different roles in the treatment of melanoma. Next, we will summarize the application of nanomaterials from the perspectives of drugs, vaccines, cellular immunity, and so on.

## 3. Nanoparticle-Based Drug Delivery for Melanoma Therapeutics

### 3.1. Nanoparticle-Mediated Chemotherapy Delivery for Melanoma Therapy

**Figure 2:**
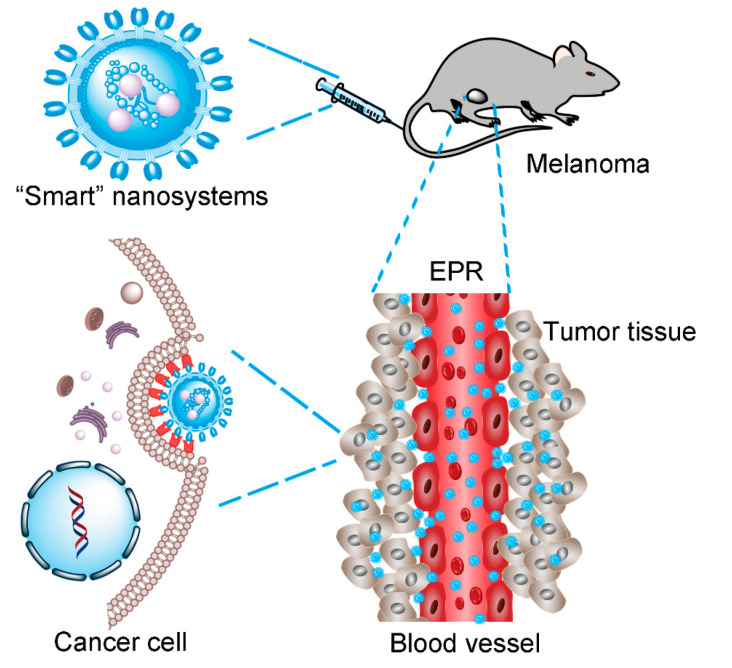
Step-wise illustration of EPR effect-based “smart” nanosystems for cancer therapy. EPR, enhanced permeability and retention effect.

A variety of antitumor drugs have been approved by the Food and Drug Administration (FDA), including DOX, ipilimumab, dabrafenib, trametinib, vemurafenib, and PTX, which have been shown to be effective against melanoma. However, the half-life of most drugs in physiological media is very short—only 1–5 h [99]. Meanwhile, it is often accompanied by severe side effects, such as allergic reactions, hypersensitivity, and severe pain. The overwhelming majority of chemotherapeutic drugs are not soluble in water or other aqueous solutions [100,101], which severely limits their efficacy in clinical applications. Considering that these chemotherapeutic drugs cannot meet the requirement of an effective drug concentration at the tumor site, researchers have used the drug delivery system (DDS) to supplement the advantages of drug utilization. In recent years, researchers have found that the nano drug delivery system can improve the bioavailability of a drug through the sustained release of the drug and prevent it from being removed by the reticuloendothelial system [102]. In addition, nano drugs can passively target tumors based on their enhanced permeability and retention effect (EPR) [103] and enhance the efficacy of chemotherapeutic drugs while reducing the systemic toxicity (Figure 2) [103]. PLGA and PEG are both FDA approved pharmaceutical excipients that are extensively used in the pharmaceutical industry. The block copolymer PEG-PLGA has long been used to fabricate PEGylated nanoparticles to overcome protein adsorption and achieve prolonged circulation following systemic administration [104]. In recent years, an increasing number of new nanocarriers has been prepared for the targeted delivery of chemotherapy drugs. For example, Nausicaa et al. demonstrated that loading PTX into a high-temperature nanosponge produced a response to several important issues associated with PTX therapy, such as solubility and toxicity [105]. The addition of PTX to nanoparticles may reduce its antitumor dose and increase the effectiveness [106]. In addition, the novel nanoparticles as PTX nanocarrier also shows a high drug loading rate, which can store and release PTX slowly and for a long time. Moreover, Zhou et al. reported PLGA nanoparticles coated with the membranes of neutrophils provided a biomimetic drug delivery system and achieved the target specificity for malignant melanoma [107]. As the most abundant leukocytes in the systemic circulation, neutrophils can be recruited at inflammatory sites under the action of the granulocyte colony stimulating factor in the tumor inflammatory microenvironment [108]. Therefore, the presence of neutrophil membranes on the surface of nanoparticles significantly improved the cell uptake efficiency of B16F10 cells, without changing the main internalization pathways. At the same time, the existence of the neutrophil cell membrane showed stronger specific aggregation at the tumor site, and the antitumor effect was stronger.

According to a recent report, the most common site of metastatic primary cancers of all types is in the lung, owing to its high vascular density [109,110]. Lung metastases can be fatal if left untreated, and there is currently no specific treatment. Systemic chemotherapy is one of the standard treatments employed for pulmonary metastasis, but its efficacy is not ideal due to its weak targeting and poor accumulation in the lung. To address this issue, Zhao et al. reported an efficient erythrocyte leveraged chemotherapy (ELeCt) platform, which is composed of biodegradable drug nanoparticles assembled on the surface of erythrocytes and can be used for chemotherapy for melanoma lung metastasis treatment. Compared with free nanoparticles, the ELeCt platform significantly prolongs the circulation time of drug nanoparticles and increases the drug content by 10-fold. In early- and late-stage melanoma lung metastasis models, the ELeCt platform can significantly inhibit tumor growth, thereby significantly improving survival [111]. In addition, various commonly used chemotherapeutic agents (e.g., DOX) can be loaded into biodegradable nanoparticles, which can be further manufactured and successfully assembled onto erythrocytes [112,113]. In summary, these studies indicate that the ELeCt platform provides a universal strategy that increases the effectiveness of chemotherapy in treating the lung metastases of melanoma.

In addition, a novel multifunctional self-transfer polymer nanoparticle was prepared to deliver the drugs that inhibit melanoma metastasis effectively. The hydrophilic portion (low molecular weight heparin (LMWH)) inhibits the last stage of the metastatic cascade by inhibiting P-selectin on activated platelets, thereby inhibiting platelet adhesion to tumor cells [114]. The hydrophobic fragment (d-α-tocopherol succinate (TOS)) can inhibit tumor resistance and increase the apoptosis of many cancer cell types, including B16F10 melanoma cells [115]. Moreover, overexpressed FA receptors on the tumor cell membrane are associated with malignant and metastatic cancer phenotypes [116,117]. Therefore, these FA-modified nanocarriers provide an effective method for the treatment of solid melanoma and metastatic tumor. In vitro and in vivo results showed that FA-modified nanoparticles with DOX significantly reduced the number of metastatic nodules without systemic toxicity [118,119]. In conclusion, clinical data indicate that the application of nanocarriers in drug delivery can effectively reduce the side effects of chemotherapeutic drugs while holding the promise of targeted therapy. However, the use of single nanocarriers for drug delivery has not yet solved the drug resistance of tumor cells, so it is necessary to study further how nanocarriers solve the problem of the drug resistance of tumor cells.

### 3.2. Nanoparticle-Based Strategies for Melanoma mRNA Vaccine Therapy

The mRNA vaccine is a promising drug for cancer prevention and treatment [120]. In contrast to DNA vaccines, mRNA vaccines can express the target protein directly, thus avoiding the side effects associated with various mutations during transcription [121]. Moreover, mRNA vaccines can be specifically designed to encode a variety of peptide and protein structures to express complete antigens [122]. In addition, an mRNA vaccine would enable us to respond more quickly to highly infectious and dangerous pandemic outbreaks, such as SARS-CoV-2 [123], and mutates in production much faster and more flexibly than existing vaccines. Currently, clinical trials of direct administration of synthetic mRNAs encoding tumor antigens have demonstrated safety, induction of tumor-specific immune responses, and some clinical benefits for patients [124]. However, mRNA delivery therapy in vivo has been considered a major bottleneck. Recent studies have shown that the incorporation of therapeutic mRNA nanoparticles can overcome in vivo delivery problems, such as the insufficient expression of intracellular proteins, and the deficient antigen loading, as well as the maturation of antigen-presenting cells [125]. In 1994, the group of Curiel et al. was the first to evaluate the effect of mRNAs on the in vivo delivery of liposomes and demonstrated that the expression of the mRNAs-cationic liposome complex was comparable to the corresponding pDNA complex when injected into tumors [126]. In 2015, liposomes were first introduced as a more advanced mRNA lipid formulation. This new mRNA formulation concept is based on previously developed pDNA, oligonucleotide, and siRNA formulation strategies. The group of Matthias et al. reported on the development of a lipid nanoparticle formulation for the delivery of mRNA vaccines to induce a cytotoxic CD 8 T cell response [127]. They confirmed that the vaccine’s effectiveness was tested in a model of B16F10 malignant melanoma. The treatment of B16F10 melanoma with lipid nanoparticles encoded with tumor-associated antigens gp100 and TRP 2 [128] mRNA resulted in tumor shrinkage and extended the overall survival in treated mice. In addition, the addition of adjuvants can further enhance the immune response. In a similar report by Yu et al., they reported a preclinical cancer vaccine that introduces both an mRNA antigen and an immune checkpoint that blocks siRNA from entering antigen-presenting cells [129]. A lipid-coated calcium phosphate (LCP) nanoparticle was used as a carrier to effectively deliver mRNA to dendritic cells (DCs) in lymph nodes for antigen expression. The LCP mRNA vaccine encoding TRP2 elicited a powerful antigen-specific cytotoxic T cell response and humoral immune response in a C57BL/6 mouse model of B16F10 melanoma. Miao et al. reported a combinatorial library of ionizable lipid-like materials to recognize mRNA delivery vehicles and facilitate mRNA delivery in vivo, and meanwhile to provide powerful and specific immune activation [130]. They also demonstrated that lipids with cyclic amino head groups activate the MYD88/RLR independent intracellular stimulator of interferon genes (STING) pathway. In addition, cyclic lipids that can activate STING are condensed with mRNA to prepare lipid nanoparticles [131]. Therefore, nanoparticle-mediated endocytosis improves the cell internalization, thereby activating the intracellular STING pathway. These agents also induce the maturation of antigen-presenting cells through the STING pathway to inhibit melanoma proliferation. In conclusion, based on current preclinical data, mRNA therapies have the potential to lead to a major revolution in medicine as they enable personalized medicine that allows tumor patients to produce their own therapeutic proteins. Furthermore, mRNA therapies will be cheaper than existing treatments because they can be produced through the gene production process and the mRNA sequence can be easily modified if needed.

### 3.3. Nanoparticle-Based Strategies for Melanoma Immunotherapy

Cancer immunotherapy is to activate or induce the patient’s host immune response to kill tumor cells [132]. In addition, the success of cancer immunotherapy depends on antigen-presenting cells, such as DCs and macrophages [133]. However, due to the influence of the tumor immunosuppressive microenvironment, DCs have been in an immature non-functional state, so their function of initiating the immune response is significantly hindered. Toll-like receptor (TLR) agonists have been reported to induce DC maturation [134], but as low molecular weight substances, most of them appear to be systematically distributed after a local injection, thus eliminating systemic proinflammatory cascading and severe immune-related toxicity. To address this issue, Wang et al. designed a drug delivery system based on mesoporous polydopamine nanoparticles and used the TLR7 agonist imiquimod (R837) as a model immunomodulator to activate immune responses of the lymph nodes [135]. Lymph nodes are important tissues of the immune system [136]. Some important immune cells (such as DC and T cells) reside in this tissue and organize the immune response. Therefore, direct delivery of R837 to lymph nodes may provide a great opportunity to increase its bioavailability and reduce side effects. The research results suggest that mesoporous polydopamine (MPDA) nanoplatforms loaded with R837 are more effective in lymphatic targeted immune stimulation for tumor immunotherapy. In addition, autophagy-regulated nanoactivators within the DCs make melanoma immunotherapy possible. A novel self-assembled nanoactivator was synthesized using a poly (β-amino ester) polymer with covalently conjugated beclin1 (NH_2_-CGTNVFNATFHIWHSGQFGT-COOH) [137] and ovalbumin (OVA, NH_2_-CSIINFEKL-COOH) [138] on both terminals of the backbone, which entered DCs and induced autophagy; the autophagy process facilitates antigen presentation and subsequent T cell activation. Yi et al. demonstrated that nanoactivators significantly enhanced tumor antigen cross-presentation and subsequent T cell initiation [139]. Moreover, in vivo experiments showed that the nanoactivators successfully reduced the number of tumors and prolonged the survival time of mice [139]. Taken together, these results suggest that nanoactivator-induced autophagy enhances the dendritic cell responses in antigen presentation to eliminate the tumors.

Previous studies have found that nanocarriers are not only used for the delivery of immune adjuvants but also have the function of activators to promote immunotherapy. Several research groups have designed iron oxide nanoparticles as nanocarrier adjuvants for dendritic cell-based cancer immunotherapy [140]. Luo et al. first synthesized ultra-small Fe_3_O_4_ nanoparticles as a nano immunoenhancer and combined them with OVA as a tumor model antigen [141]. Interestingly, free Fe_3_O_4_ nanoparticles showed significant immunotherapeutic ability in these experiments, demonstrating that Fe_3_O_4_ nanoparticles not only serve as a delivery tool to protect antigens from degradation and inactivation but also participate in cancer immunotherapy as an enhancer to promote immune responses [142]. The experimental results demonstrated that the therapeutic and preventive effect of the Fe_3_O_4_-OVA vaccine on subcutaneous or metastatic melanoma growth and formation was demonstrated to be based on dendritic cell immunotherapy and potential macrophage activation. Recently, Chen et al. developed a new sensitizer, copper cysteamine (Cu-Cy), which was evoked by UV, X-rays, microwaves, and ultrasound to produce reactive oxygen species (ROS) to destroy cancer cells and bacteria [143]. Zhang et al. designed Cu-Cy nanoparticles with an average size of ~40 nm to produce substantial levels of ROS and promote the apoptosis and/or necrosis of melanoma cells under X-ray stimulation [144]. Furthermore, this binding promotes the formation of an antitumor immune response. These results suggest that Cu-Cy nanoparticles can simultaneously achieve radiotherapy, oxidation therapy, and immunotherapy for tumors, and help overcome the limitations of traditional melanoma treatment strategies.

T cell therapy has been a great success in the treatment of hematologic malignancies [145]. However, T cells have had limited success in treating solid tumors. Most researches focus on the inhibitors antagonizing the proinflammatory cytokines or immune checkpoint to improve efficacy [146,147]. However, some patients have unsatisfactory therapeutic effects and serious side effects [148]. Hence, it is necessary to develop effective and safe combined treatment methods. Previous studies have shown that the tumor-specific microenvironment inhibits T cell infiltration, survival, and effector function [149,150]. In addition, studies have shown that T cell function depends on cholesterol in the cell membrane to gather T cell receptors (TCR) and form immune synapses. Therefore, it is expected to improve the immunotherapy of solid tumors by regulating cholesterol metabolism in combination with T cell therapy. Avasimibe (Ava) is an inhibitor of the cholesterol esterification enzyme acetyl-CoA acetyltransferase 1. It increases the plasma membrane cholesterol content, thereby promoting TCR aggregation and improving the T cell function [151]. Hao et al. used click chemistry to attach Ava-containing nanoliposomes to the surface of engineered T cells without interfering with their physiological functions [26]. Finally, studies have shown that TCR transgenic CD8^+^ T cells and chimeric antigen receptor T cells carrying liposomal AVA have good antitumor effects in mouse models of melanoma and glioblastoma [152].

### 3.4. Nanoparticle-Based Strategies for Melanoma Photodynamic Therapy (PDT)

Nanotechnology is expected to play an important role in modern cancer treatment, including primary and metastatic melanoma. Nanoparticles are capable of targeting and visualizing the transfer to a variety of organs and delivering therapeutic drugs. By targeting tumor cells, nanoparticles can greatly reduce the minimal drug toxicity to healthy tissues/organs [153]. Due to its controllability and traceability, nanotechnology-based PDT [154,155] and thermotherapy [156] may be a powerful method for cancer research and treatment in the future. The early treatment of melanoma is effective, and diagnosis is necessary before metastasis occurs. However, early diagnosis of tumors is a challenge for clinicians and scientists because clinical symptoms appear at a later stage of the tumor. Therefore, a novel type of early diagnosis nanomaterial needs to be developed to provide earlier and more accurate tumor detection.

In addition to traditional chemical drugs and gene drugs, PDT for melanoma treatment has emerged and developed in recent years [157]. PDT is a typical treatment strategy with specificity, low drug resistance, and high temporal and spatial precision [158]. PDT consists of three components: light, photosensitizer (PS), and tissue oxygen. During the PDT treatment of cancer, PS transfers its excitation energy to surrounding oxygen molecules to generate ROS, such as superoxide anion radicals [159], hydroxyl radicals [160], hydrogen peroxide radicals [161], and singlet oxygen [162], to induce cancer cell death. PDT can induce platelet aggregation and the release of vasoactive molecules, increase the vascular permeability and vasoconstriction, and lead to damage of the vascular system around tumor tissue, thereby inhibiting tumors. Some clinical reports have shown the efficacy of PDT in the treatment of patients with metastatic melanoma [163,164]. However, traditional PS molecules, such as porphyrins [165] and chlorin e6 (CE6) [166], which are commonly used as PDT PSs for image-guided cancer therapy, have many defects, including a low solubility and fluorescence quantum yield, and aggregation and quenching. To address this issue, Cheng et al. proposed a self-assembled delivery system for the PDT treatment of malignant melanoma [167]. The self-assembled nanocarrier system consists of the PS protoporphyrin, a melanoma-specific antigen peptide (KVPRNQDWL) [168], and PEG to achieve preferred tumor accumulation by EPR. PDT can enhance immunogenicity, improve the efficiency of antigen cross-presentation, and form more effective tumor-specific cytotoxic T cells. In addition, the melanoma-specific antigen peptide, which is a melanoma-specific antigen delivered in a nanocarrier, can also activate specific cytotoxic T cells to achieve durable antitumor immunity. Furthermore, Chien et al. developed a method for treating melanoma using the free form and micellar form of the PS CE6 in PDT [169]. Compared with the free CE6, the micellar nanocarrier attached CE6 had a clearer vascular image, and the micellar CE6 was localized in the lysosome and endoplasmic reticulum of cultured endothelial cells, suggesting active endocytosis of the nanocarrier. In summary, micellar CE6 potentially functions as a dual-function PS for angiography and PDT to promote their delivery in the tumor microenvironment. At the same time, Li et al. reported liposome encapsulated aggregation induced emission iuminogen nanoparticles (AIE nanoparticles) [170]. Under near-infrared light (800 nm), a high quantum yield (23%) and a maximum two-photon absorption (TPA) cross section of 560 GM were observed. The research results show that AIE nanoparticles can be used as imaging agents for the spatiotemporal imaging of tumor tissues, and the penetration depth of mouse melanoma models can reach 505 μm. Although PDT is a promising treatment for melanoma, its application in the treatment of malignant melanoma has not been fully promoted. The most important limiting factor is that the antioxidant effect of melanin may inhibit PDT in the treatment of melanoma. In addition, the hypoxic environment of tumors further limits the application of PSs in tumor therapy [171]. Therefore, researchers hope to improve the efficacy of PDT in the treatment of melanoma by improving the hypoxic environment. Additionally, SiO_2_ [172], Fe_3_O_4_ [173], and mesoporous titanium dioxide (TiO_2_) [174] are not only a carrier, but also a potential PS. Zhou et al. proposed a kind of TiO2 as a PS and doped with MnO2 to form core-shell MnO2@TiO2 nanoparticles (MTM nanoparticles) [175]. MTM nanoparticles can provide oxygen sustainably, thereby increasing the ROS level and reducing tumor hypoxia and tumor metastasis. These studies provide a potential strategy for high brightness, superior photostability, and high biosafety nanomaterial-based tumor imaging system. Such a system will benefit PDT therapy in the near future.

### 3.5. Combination Therapy

Based on previous medication, we found that a single drug treatment for early cancer may show good efficacy, but with a continuation of the treatment time, tumor cells often develop resistance, which means that patients have to change to new drugs for treatment, and a new drug can bring more potential side effects to patients and the cost of expensive treatment. The combination of two or more anticancer drugs with different antitumor mechanisms can enhance the therapeutic effect, reduce adverse reactions, and prevent the occurrence of drug resistance through the synergistic effect between different drugs [176]. However, due to the differences in the physical and chemical properties of the drugs, it is difficult for the drugs to be enriched in the tumor tissue at the same time, which severely limits the clinical efficacy of the combined administration. To solve this problem, Xiong et al. designed a self-assembled nanocarrier to encapsulate cisplatin (CDDP) and metformin (MET) for co-delivery to non-small cell lung cancer [177]. Similarly, Li et al. used cationic liposomes to co-deliver both DOX and MET for treating multi-drug-resistant breast cancer cells-MCF7/ADR [178]. The faster release of MET enhances the cytotoxicity of DOX by reducing hypoxic stress in vivo and in vitro. MET diminished the cell oxygen consumption and inhibited the expression of HIF1α and P-glycoprotein (Pgp) in vitro [179]. In addition, the dual-drug loaded liposomes increased tumor targeting and intratumoral blood oxygen saturation, indicating that the tumor reoxygenation effect of MET promotes its synergistic effect with DOX to combat MCF7/ADR xenografts. Moreover, Tham et al. reported a mesoporous nano vehicle with dual loading of PSs and clinically relevant drugs for combination therapy while utilizing microneedle technology to facilitate their penetration into deep skin tissue [180]. The PSs were synthesized by covalently binding to a silica matrix, which significantly improved the quantum yield and photostability of these PSs. The mesopores of the nanoparticles were further loaded with small molecule inhibitors (dabrafenib and trametinib) that target the hyperactive mitogen-activated protein kinase (MAPK) pathway for melanoma treatment. Empty nanocarriers were biocompatible with skin cells, while near infra-red (NIR) irradiated drug-loaded nanocarriers have synergistic killing effects on skin cancer cells mainly through ROS and caspase-activated apoptosis. Recently, Liu’s team combined PDT and photothermal therapy (PTT) with chemotherapy (so-called “chemo-phototherapy”) to develop light-triggered biomimetic nanoerythrocytes for combined therapy of malignant melanoma lung metastases against tumors [181]. Under NIR laser irradiation, the erythrocyte (RBC) membrane vesicle was disrupted to trigger the rapid release of the encapsulated drug. And then, the released 1,2-diaminocyclohexane-platinum (II) (DACHPt) internalized to cancer cells by inducing DNA damage to inhibit tumor cell replication. Meanwhile, the released indocyanine green (ICG) was able to penetrate the tumor and produce more cytotoxic singlet oxygen and heat to induce tumor cell apoptosis.

Furthermore, combination therapy can better balance immune activation and suppression signals, which has great potential in cancer immunotherapy. In the process of immunogenic cell necrosis, calreticulin (CRT) transfers to the cell membrane to promote the recruitment, recognition, and antigen expression of DC to strengthen the host immune response. It has been reported that some chemotherapeutic drugs (DOX, oxaliplatin) and PDT can induce immunogenic cell death (ICD) [182,183,184]. Recent studies have shown that manganese (Mn) can enhance cyclic guanosine monophosphate/adenosine monophosphate (GMP-AMP) synthase and STING activation of viral infection [185]. Hou et al. prepared an innate immune nanoactivator to improve the efficacy of cyclic GMP-AMP synthase (cGAS)/STING pathway immunotherapy by synthesizing nanoscale amorphous porous manganese phosphate (APMP) [186]. DOX was encapsulated in APMP nanoparticles and then coated with phospholipid (PL) in APMP nanoparticles to obtain PL/APMP-DOX. The results suggest that PL/APMP-DOX nanoparticles can promote DC maturation and natural killer cell (NK) recruitment. At the same time, it activates the downstream pathway of cGAS/STING to regulate the expression of TNF-α, thereby exerting a potential anticancer effect. In conclusion, the combination of immunotherapy, chemotherapy, and PDT is emerging as a promising new treatment for cancer. However, the main challenge is to ensure that both cancer and immune cells are targeted and specifically targeted in a safe manner. Therefore, researchers need to develop new nanocarriers to address these issues.

## 4. Conclusions

Currently, nanoparticle-based treatment strategies are at the forefront of clinical research and are used to revolutionize the treatment of diseases. Specifically, in cancer, recent advances in tumor biology and nanotechnology interactions have led to multiple effective therapies in fundamentally different tumor models. At present, the drug-delivery systems (DDS) have made significant progress in formulation preparations while achieving more precise treatment at the molecular level, thus broadening the ways for personalized tumor therapy. The DDS increases the drug solubility, improves bioavailability, and prolongs the circulating time. It also selectively releases the drug at the ideal site through tissue- or cell-dependent targeted modification. Therefore, constructing an effective DDS for melanoma treatment is of great clinical significance.

Until now, liposomes, polymer nanoparticles, inorganic nanoparticles, and other systems have been widely used in the treatment of melanoma. However, pitfalls and caveats for different types of nanocarriers still exist. For example, liposomes are known to encapsulate hydrophobic and hydrophilic drugs [187] and are extensively applied in drug delivery because of their advantages with prolonged efficacy, reduced toxicity, and tumor-targeting properties after modification. However, many liposomal preparations were unexpectedly withdrawn in clinical trials, even though they are very successful in previous animal models [188]. It should be noted that most in vivo studies use a mouse solid tumor model, which overestimates the therapeutic efficacy of liposomes modified by targeting ligands. In addition, the subcutaneous tumor model grows rapidly, and its blood vessels are leaky, leading to an overestimation of the EPR effect [189]. Furthermore, the limitations of liposomes include rapid metabolism and degradation, as well as instability and difficulty in storage [187]. On the other hand, polymer nanoparticles have poor stability in physiological environments and may exchange with other physiological components, restricting their applications. Inorganic nanoparticles mainly include gold nanoparticles, iron oxide nanoparticles, silica nanoparticles, etc. [190]. Inorganic nanoparticles have been attractive due to their large-scale speed synthesis, controllable particle size, and easy surface modification. However, their metabolism and potential toxicity limit the clinical application [191]. For example, gold nanoparticles are generally considered to be non-cytotoxic, and their small particle size is conducive to rapid excretion by the kidneys. However, some studies have shown that the large accumulation of gold nanoparticles in cells causes mitochondrial toxicity and promotes cell apoptosis and necrosis. In addition, these gold nanoparticles lead to tissue apoptosis and acute inflammation when they are significantly accumulated in the liver. Last but not the least, a traditional method-based synergistic process of inorganic nanomaterials unavoidably increases the reducing agents and stabilizers on the surface, which are known to be toxic [192].

Unfortunately, complex physiological and physical barriers and adverse patient responses, safety, and effectiveness are still the biggest challenges for the clinical application of nanosystems. Researchers can find answers to these questions using carefully designed nanotechnology and platforms. Nanotechnology, especially nanomaterials, can not only be used for targeted drug delivery but can also trigger the immune system to use the patient’s own immune defense capabilities to achieve internal anticancer effects. These methods show that multifunctional nanoparticles and biomaterials have the potential to solve some of the most pressing challenges in cancer treatment. Nanoparticles extend the blood circulation time of the drug and increase the drug concentration in the lesion through polymer modifications. According to the tumor microenvironment, designing sensitive materials will achieve specific drug release results. In addition, the co-delivery of multiple drugs also overcomes the difficulty that drugs are challenging to administrate at the same time and reduces the resistance and side effects of a single drug. Moreover, a real-time tracking system can be designed by biological probes. However, so far, regulatory agencies have not approved nanomaterials for cancer treatment, and the many major deficiencies that need to be addressed. Although some nanoparticles show encouraging results, lack of bioavailability, clearance from the body, and postpartum side effects are the key shortages.

Some future challenges urgently need to be considered. First, nanoparticles and biomaterials with multiple payloads have complex properties and are targeted or activated. In addition, the complex ratio of inert excipients will complicate large-scale production, leading to repeatability problems. Although nanoparticles capable of targeted drug delivery, immune cell activation, and early diagnosis are attractive, the effective use of these combined nanomaterials requires more biomarkers to monitor responses. Therefore, it is necessary to study the short-term and long-term toxicity of these multifunctional nanomaterials, especially considering that the activation of different components of the host immune system may cause long-term immune-related side effects. In conclusion, future therapeutic strategies based on different nanocarriers through a combination of drug delivery provide hopes for the clinical melanoma treatment.

## Figures and Tables

**Table 1:** Multifunctional nanoparticles-based treatment of melanoma reported in the last three years (2018–2020).

Nanocarrier	Anticancer Agent	Functionalization	Ref
Liposome	Anacardic acid, Mitoxantrone, Ammonium ascorbate, PTX, TRAIL, Vemurafenib, Hypericin, n-Butylidenephthalide, Hydroxychloroquine, eIF3i shRNA, Hispolon, and 5-fluorouracil.	Stearyl chain (C18) fused pH-sensitive cell-penetrating peptide (C18-TR), Peptide: TD (ACSSSPSKHCG), DPPC, R8-dGR peptide, iRGD.	[82,83,84,85,86,87,88]
Polymeric nanoparticles	Indocyanine green, Pyrazoline, Dox, Gadopentetic acid, PTX, Ellagic acid, Curcumin, Antigenic peptide (hgp10025-33), and TLR 4 agonist.	Proteins, Chitosan, Cyclodextrin and Starch, PLGA, RBC.	[89,90,91,92]
Dendrimer	Cytosine–phosphate–guanine oligonucleotides and DOX.	PEG-PAMAM, LMWH.	[93,94]
Carbon nanoparticles	-	Fluorescent nitrogen–phosphorous-doped carbon dots.	[95]
Metal nanoparticles	Dacarbazine, Mesilato de imatiniband siRNA STAT-3 and Cas9-sgPlk-1 plasmids.	Magnetic nanoparticles, Gold nanoparticles, and Silica nanoparticles.	[96,97,98]

PTX, Paclitaxel; TRAIL: Tumor necrosis factor (tnf)-related apoptosis inducing ligand; eIF3i: Eukaryotic translation initiation factors 3i; DOX: Doxorubicin; TLR: Toll-like receptor; STAT-3: Signal Transducer and Activation of Transcription 3 factor; DPPC: Dipalmitoylphosphatidylcholine; iRGD: internalizing RGD peptide; PLGA: Poly (d, l-lactic-coglycolic acid); RBC: Erythrocyte (RBC); PAMAM: Poly(ethylene glycol)-polyamidoamine; LMWH: Low-molecular-weight heparin.
